# MRE11 Deficiency Occurs in a Small Group of Cancers from Various Different Tumor Entities †

**DOI:** 10.3390/diagnostics16131965

**Published:** 2026-06-24

**Authors:** Viktor Reiswich, Henry Recksiek, Katharina Möller, Florian Lutz, Florian Viehweger, Georgia Makrypidi-Fraune, Martina Kluth, Claudia Hube-Magg, Christian Bernreuther, Guido Sauter, Andreas H. Marx, Ronald Simon, Till Krech, Stefan Steurer, Christoph Fraune, Sarah Minner, Viktoria Chirico, Veit Bertram, Clara Lühr, Cosima Völkel, Morton Freytag, Natalia Gorbokon, Maximilian Lennartz, Eike Burandt, Anne Menz, Clara von Bargen

**Affiliations:** 1 Institute of Pathology, University Medical Center Hamburg-Eppendorf, 20246 Hamburg, Germanya.menz@uke.de (A.M.);; 2 Department of Pathology, Academic Hospital Fuerth, 90766 Fuerth, Germany; 3 Institute of Pathology, Clinical Center Osnabrueck, 49076 Osnabrueck, Germany

**Keywords:** MRE11-deficient, human cancers, immunohistochemistry, tissue microarray

## Abstract

**Background/Objectives**: The double-strand break repair protein MRE11 forms the core of the MRE11/RAD50/NBS1 (MRN) complex. Cancers with reduced MRE11 expression have been suggested to be more sensitive to radio-chemotherapy and may be subject to synthetic lethality. The aim of this study was to assess the prevalence of MRE11 deficiency and the potential role and clinical significance of elevated and/or reduced MRE11 expression in human cancer. **Methods**: A tissue microarray containing 14,966 samples from 134 different tumor entities was analyzed for MRE11 by immunohistochemistry. **Results**: In normal tissues, strong nuclear MRE11 staining occurred in almost all cell types. In cancers, nuclear MRE11 staining was strong in 11,797 (91.0%), moderate in 1018 (7.9%), weak in 86 (0.7%), and completely absent (MRE11 deficiency) in 55 (0.4%) of 12,956 informative tumor samples. Only six tumor entities had more than one MRE11-deficient cases including hepatocellular carcinoma (9 of 193), intestinal type gastric adenocarcinoma (4 of 208), endometrioid endometrial carcinoma (5 of 268), pulmonary adenocarcinoma (2 of 165), colorectal adenocarcinoma (CRC, 16 of 2183), and clear cell renal cell carcinoma (ccRCC, 7 of 1011). Reduced MRE11 staining was associated with mismatch repair deficiency (dMMR) in CRC and in gastric adenocarcinoma (*p* < 0.0001 each), advanced pT stage (*p* = 0.0003) and L1 status (*p* = 0.0019) in testicular seminoma, high grade (*p* < 0.05), advanced pT (*p* < 0.0001), and high UICC stage (*p* = 0.0014) in ccRCC, advanced pT stage in high-grade serous ovarian carcinoma (*p* = 0.0396), and nodal metastases in papillary thyroid cancer (*p* = 0.0332). **Conclusions**: MRE11 is highly expressed in most cancers. Reduced MRE11 expression is associated with aggressive phenotype in multiple cancer types. The potential to exploit MRE11 deficiency as a target for synthetic lethality deserves to be further explored.

## 1. Introduction

The double-strand break repair protein MRE11 forms the core of the MRE11/RAD50/NBS1 (MRN) complex which plays a pivotal role in the repair of double-strand DNA breaks (DSBs) [1,2]. The MRN complex acts as an early DNA damage response (DDR) element which can sense DSBs, recruit further DDR proteins and activate their downstream signaling, and contribute to DSB repair through its single-stranded DNA endonuclease and 3′ to 5′ exonuclease activity [3]. The MRN complex is not only involved in homologous recombination (HR) of DSBs but also plays a role in non-homologous end joining (NHEJ) and the more error-prone pathway of microhomology-mediated end-joining (MMEJ) repair for which MRE11 constitutes one of six required enzymes [4,5].

The role of MRE11 in cancer is complex and not fully understood. While MRE11 is expressed at significant levels in virtually all normal cell types, altered expression has been described in various tumor entities including prostatic [6], colorectal [7,8], and gastric adenocarcinoma [9,10], breast cancer [11,12], squamous cell carcinoma of the esophagus [13], and in urothelial carcinoma of the urinary bladder [14,15,16]. High MRE11 expression was found to be linked to unfavorable tumor features in several of these tumor entities [6,9,10,11] while other authors described associations between low MRE11 expression and aggressive disease in breast cancer [12] and in urothelial carcinoma of the urinary bladder [11]. Most of the clinical interest in MRE11 and the MRN complex comes from studies emphasizing a predictive role of MRE11 expression. MRE11-overexpressing cancers have been described to be more radioresistant while low-expressors were more sensitive to radiation and chemotherapy with camptothecin and gemcitabine [17,18,19,20]. Several MRE11 inhibitors are currently investigated in clinical trials as radiosensitizers [21]. Given the pivotal role of MRE11 in DNA repair and genomic instability, synthetic lethality may occur in cancers where MRE11 is defective and alternative pathways are druggable [22]. For example, experimental studies have shown that MRE11-deficient cancer cells showed a markedly increased susceptibility to treatment by PARP inhibitors [23].

The aim of this study was to assess the prevalence of MRE11 deficiency and the potential role and clinical significance of elevated and/or reduced MRE11 expression in human cancer. Therefore, more than 14,000 tissue samples from 134 different tumor types and subtypes, and 76 non-neoplastic tissues were evaluated by immunohistochemistry (IHC) for MRE11 expression in a tissue microarray (TMA) format.

## 2. Materials and Methods

### 2.1. Tissue Microarrays (TMAs)

Our normal tissue TMA was composed of 8 samples from 8 different donors for each of 76 different normal tissue types (608 samples on one slide). The cancer TMAs contained a total of 14,966 primary tumors from 134 tumor types and subtypes. Detailed histopathological and molecular data were available for 1224 clear-cell and 310 papillary renal-cell carcinomas (ccRCC and pRCC), 2351 colorectal (CRC) and 327 gastric adenocarcinomas, 1680 invasive breast cancers of no special type (NST), 598 ductal adenocarcinomas of the pancreas, 231 hepatocellular carcinomas, 369 serous high-grade carcinomas of the ovary, 182 endometrioid carcinomas of the endometrium, 565 testicular seminomas, and 382 papillary carcinomas of the thyroid. The full spectrum of tumor stages and grades are included to limit the impact of a specific tumor stage or grade on the prevalence of MRE11 deficiency. The composition of both normal and cancer TMAs is described in detail in the Section 3. All samples were from the archives of the Institute of Pathology, University Medical Center Hamburg-Eppendorf, Germany; the Institute of Pathology, Clinical Center Osnabrueck, Germany; and Department of Pathology, Academic Hospital Fuerth, Germany. Tissues were fixed in 4% buffered formalin and then embedded in paraffin. TMA tissue spot diameter was 0.6 mm. The TMA manufacturing was carried out at the Institute of Pathology of the University Medical Center Hamburg-Eppendorf as previously described [24,25]. The usage of archived diagnostic left-over tissues for manufacturing of TMAs and their analysis for research purposes as well as patient data analysis has been approved by local laws (HmbKHG, §12,1) and by the local ethics committee (Ethics commission Hamburg, WF-049/09; date of approval: 25 November 2010). All work has been carried out in compliance with the Helsinki Declaration.

For the comparison with previously published IHC studies on MRE11 expression (Supplementary Figure S2), a PubMed search was performed using the terms “MRE11”, “cancer” and “immunohistochemistry”.

### 2.2. Immunohistochemistry

Freshly cut TMA sections were immunostained on one day and in one experiment. All slides were processed in parallel using the same lot of primary antibody and identical incubation conditions, antigen retrieval and detection protocols to ensure direct comparability across samples. Slides were deparaffinized with xylol, rehydrated through a graded alcohol series and exposed to heat-induced antigen retrieval for 5 min in an autoclave at 121 °C in pH 7.8 Tris-EDTA-Citrat (TEC) puffer. Endogenous peroxidase activity was blocked with Dako REAL Peroxidase-Blocking Solution (Agilent Technologies, Santa Clara, CA, USA; #S2023) for 10 min. Primary antibody specific for MRE11 (recombinant rabbit monoclonal, HMV328, ardoci GmbH, Hamburg, Germany) was applied at 37 °C for 60 min at a dilution of 1:150. For the purpose of antibody validation, the normal tissue TMA was also analyzed by the polyclonal rabbit MRE11 antibody #4895 (Cell Signaling Technology, Leiden, The Netherlands) at a dilution of 1:450 and an otherwise identical protocol. Bound antibody was then visualized using the EnVision Kit ™ (Agilent, CA, USA; #K5007) according to the manufacturer’s directions. The sections were counterstained with haemalaun. For tumor tissues, the staining intensity of tumor cells was semi-quantitatively recorded as 0, 1+, 2+, or 3+. Presence of unequivocal MRE11 positivity in stroma cells was required to classify a tumor as “0” (MRE11 completely negative, MRE11 deficiency). Tumors lacking MRE11 staining in both tumor and stroma cells were categorized as “non-informative”.

### 2.3. Statistics

Statistical calculations were performed with JMP^®^ 18 software (SAS Institute Inc., Cary, NC, USA). Contingency tables and the chi^2^-test were performed to search for associations between MRE11 immunostaining and tumor phenotype.

## 3. Results

### 3.1. Technical Issues

A total of 12,956 (86.6%) of 14,966 tumor samples were interpretable in our TMA analysis. Non-interpretable samples were due to absence of MRE11 staining in both tumor and stroma cells, a lack of unequivocal tumor cells, or a lack of entire tissue spots. A sufficient number of samples (≥4) of each normal tissue type was evaluable.

### 3.2. MRE11 in Normal Tissues

**Figure 1:**
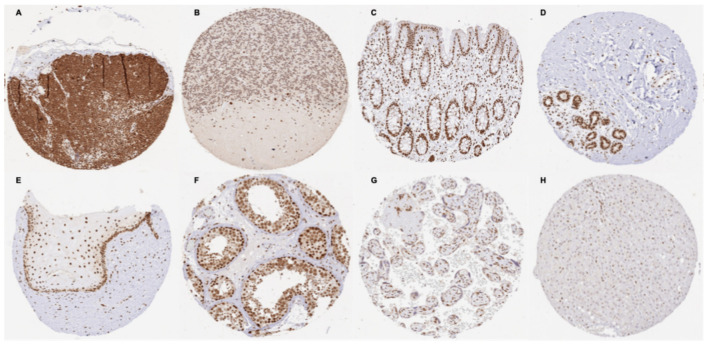
MRE11 immunostaining of normal tissues. The panels show a nuclear MRE11 immunostaining of all cells in the thymus although a small subset of cells stands out because of their even stronger positivity (**A**), a rather weak MRE11 staining in the cerebellum with a markedly stronger staining intensity in Purkinje cells (**B**), and a generally strong MRE11 staining in the rectum (**C**), breast (**D**), and squamous epithelium of cervix uteri (**E**). The intensity of MRE11 staining decreases with maturation in cells of the spermiogenesis in the testis (**F**), and it is low or absent in syncytiotrophoblastic cells of the mature placenta (**G**) and in hepatocytes (**H**). Staining was performed with the antibody HMV328, ardoci GmbH, Hamburg, Germany by immunohistochemistry. Image magnification: 40×.

A strong nuclear MRE11 staining was observed in the vast majority of cell types. It was particularly strong in a dispersed (probably epithelial) cell type of the thymus and in Purkinje cells of the cerebellum. As compared to most other cell types, MRE11 staining was weaker in maturing cells of the spermiogenesis, superficial cell layers of non-keratinizing squamous epithelium, and in syncytiotrophoblasts of the mature placenta. Nuclear MRE11 staining was also reduced in hepatocytes of the liver where it was even absent in a fraction of the samples. Representative images are shown in Figure 1.

All these nuclear stainings, including subtle differences in staining intensity, were seen by both HMV328 and the polyclonal antibody #4895. An additional cytoplasmic staining of parietal cells of the gastric mucosa was only seen by HMV328 while additional cytoplasmic stainings of hepatocytes, testicular Leydig cells, and a subset of adrenocortical cells were only observed by #4895 antibody (Supplementary Figure S1). Some cytoplasmic staining was also seen by both antibodies in some smooth muscle cells, trophoblast cells of the first trimester placenta, and a tubular cell type of the testis.

### 3.3. MRE11 in Cancer Tissues

A nuclear MRE11 staining occurred in all cells of the vast majority of cancers. Of 12,956 evaluable cancers, only 55 (0.4%) showed a complete MRE11 expression loss (MRE11 deficiency) while 86 (0.7%) showed a weak, 1018 (7.9%) a moderate, and 11,797 (91.0%) a strong MRE11 positivity (Table 1).

**Figure 2:**
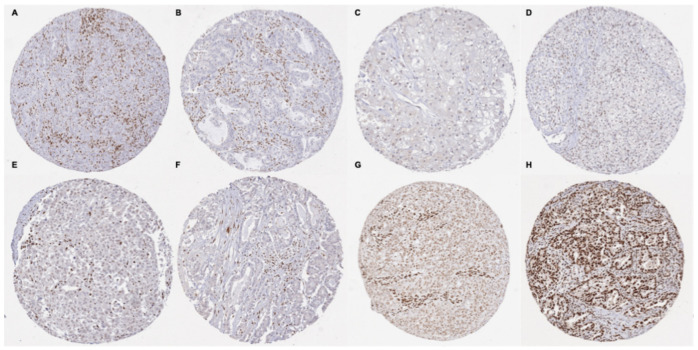
MRE11 immunostaining in cancer. The panels show a loss of MRE11 staining in cancer cells of two adenocarcinomas of the colon (**A**,**B**), two clear cell renal cell carcinomas (**C**,**D**), an endometrioid endometrial carcinoma (**E**), and a gastric adenocarcinoma (**F**), while a distinct and often strong nuclear MRE11 staining occurs in stromal cells of all these samples. Nuclear MRE11 immunostaining was weak in an endometrioid carcinoma of the ovary (**G**) and strong in a pulmonary adenocarcinoma (**H**). Staining was performed with the antibody HMV328, ardoci GmbH, Hamburg, Germany by immunohistochemistry. Image magnification: 40×.

More than one case with MRE11 deficiency was only seen in six tumor entities including hepatocellular carcinoma (9 of 193; 4.7%), gastric adenocarcinoma of intestinal type (4 of 208; 1.9%), endometrioid endometrial carcinoma (5 of 268; 1.9%), pulmonary adenocarcinoma (2 of 165; 1.2%), CRC (16 of 2183; 0.7%), and ccRCC (7 of 1011; 0.7%). One case each with a MRE11 deficiency had been found in 11 further tumor entities. Representative tumor images are shown in Figure 2.

Given the low number of MRE11-deficient cases, their statistical associations with phenotype could not be calculated. Because of the high number of cases with “strong” positivity, tumors were categorized as “strongly positive” vs. “low to intermediate positive”. Low-to-intermediate (reduced) staining was associated with mismatch repair deficiency (dMMR) and right side tumor location in CRC (*p* < 0.0001 each; Table 2), dMMR in gastric adenocarcinoma (*p* < 0.0001), advanced pT stage (*p* = 0.0003) and L1 status (*p* = 0.0019) in testicular seminoma, high grade (*p* < 0.05), advanced pT stage (*p* < 0.0001), and high UICC stage (*p* = 0.0014) in ccRCC, advanced pT stage in high-grade serous ovarian carcinoma (*p* = 0.0396), and nodal metastases in papillary thyroid cancer (*p* = 0.0332).

The MRE11 expression level was unrelated to clinical, histopathological and/or molecular features in 400 invasive breast cancers of no special type (NST), 166 endometrioid endometrium carcinomas, 258 pRCCs, and 477 ductal pancreatic adenocarcinomas.

## 4. Discussion

Because MRE11 is expressed at high levels in all cell types of the tumor stroma, RNA data have little utility for assessing the role of MRE11 expression in cancer, and IHC represents the best option to analyze MRE11 expression in tumor cells. Considering the large dimension of our study, a particularly thorough validation of our IHC assay was performed. Following the recommendations of the International Working Group for Antibody Validation (IWGAV) [26], our assay was validated by confirming our IHC staining by a second, independent antibody on 76 different categories of normal tissues. These experiments confirmed the specificity of the nuclear staining of our assay because similar staining patterns were observed by both HMV328 and #4895 antibodies, including identical high-level staining in dispersed cells of the thymus and in Purkinje cells of the cerebellum as well as low-level staining in maturing cells of the spermiogenesis, hepatocytes of the liver, superficial cell layers of non-keratinizing squamous epithelium, and syncytiotrophoblasts of the mature placenta. The additional cytoplasmic staining of parietal cells of the stomach by HMV328 and of hepatocytes, testicular Leydig cells, and of adrenocortical cells by #4895 were considered (tolerable) cross-reactivities of the individual antibodies. Because of its cytoplasmic nature, cytoplasmic staining in some smooth muscle cells, trophoblast cells of the first trimester placenta, and a tubular cell type of the testis was also regarded as non-specific although it was seen by both antibodies. It is of note that the use of a very broad range of different tissues (76 different normal tissue categories) for antibody validation increases the likelihood for detecting undesired cross-reactivities because virtually all proteins (and their post-translational modifications) occurring in normal cells of adult humans are subjected to the validation experiment.

Our observation of a complete MRE11 loss (MRE11 deficiency) in 55 (0.4%) of our tumors may represent the clinically most significant finding of our study. Our data suggest that tumor entities with the highest likelihood for being MRE11-deficient include hepatocellular carcinoma (4.7%), intestinal-type gastric adenocarcinoma (1.9%), and endometrioid endometrial carcinoma (1.9%). Although the overall frequency of MRE11 deficiency is rather low, our findings could be of considerable clinical significance. MRE11 plays a critical role in multiple pathways of DNA damage recognition and repair, and the maintenance of genomic stability. It is therefore expected that MRE11-deficient cells may be more and perhaps critically dependent on alternative repair pathways some of which may be druggable by small molecule inhibitors to result in synthetic lethality [3,4,22,27,28]. Several in vitro studies have indeed shown that loss of MRE11 expression increases sensitivity to PARP inhibitors in colorectal [23], hematological [29] and endometrial cancer cell lines [22,30]. Loss or inhibition of MRE11—or RAD50 and NSB1—impairs the functionality of the MRN complex and the cell capacity for DSB repair [3]. A simultaneous inhibition of PARP-driven repair of single strand breaks (SSBs) results in an accumulation of SSBs in proliferating cells, which can result in replication forks stalling and collapse and consequently the formation of DSBs which cannot be adequately repaired by MRE11-deficient cells. In line with these observations, synthetic lethality has been described in vitro for dual inhibition of PARP and MRE11 [27]. It is also of note, that low MRE11 expression has been reported to predict a favorable response to radiotherapy [16,21,31] and cytotoxic drugs such as camptothecin and gemcitabine [19,32,33].

As the identification of cancers with MRE11 deficiency was the main aim of this study, our IHC protocol was designed to be as sensitive as possible to avoid “false negative” staining results. This approach resulted in a very high number of cases with high (3+) expression levels and a markedly higher rate of MRE11 positivity than in the 22 studies which had earlier evaluated MRE11 expression by IHC in cancer cohorts (summarized in Supplementary Figure S2). That tumors with less than 3+ MRE11 positivity tended to exhibit prognostically unfavorable histopathological tumor features in seminoma, ccRCC, high-grade serous ovarian cancer, and papillary thyroid cancer is in line with data from several other studies. Significant associations between low MRE11 expression and histopathological or clinical parameters of cancer aggressiveness have been described for breast [34] and ovarian cancer [35]. Considering the cellular function of MRE11, it could be assumed that aggressive cell behavior of cancer cells with low MRE11 expression could be caused by compromised DNA repair and consequent genomic instability [30,36]. In line with this notion, reduced MRE11 expression has been shown to impair cGAS activation, which is essential in response to oncogenic stress, cytosolic dsDNA and ionizing radiation [37]. While it is also possible that a loss of MRE11 in aggressive cancers might just reflect random loss of protein expression as a result of tumor cell dedifferentiation paralleling cancer progression, it appears counterintuitive that a loss of a critical DNA repair protein would not result in a notable functional effect in cancer cells. The strong association between low MRE11 expression and dMMR in both CRC and gastric adenocarcinoma is in agreement with data from several earlier studies [23,38,39]. This strong link could be explained by dMMR/microsatellite instability (MSI)-driven functionally relevant mutations in the microsatellite tract repeats of the MRE11 gene—for example the intronic MRE11 poly(T)11 region—that result in a reduced or absent MRE11 expression [23,39,40].

It is noteworthy that several previous IHC studies have reported a significant relationship between high MRE11 expression and poor prognosis or prognostically unfavorable histopathological tumor features in breast cancer [11], prostatic [6], gastric [9,10], and colorectal [17] adenocarcinoma, urothelial carcinoma [15], and in squamous cell carcinoma of the oral cavity [41]. Our data did not suggest a major unfavorable prognostic impact of high MRE11 expression. However, the group of 3+ cases was very large in most of our tumor categories due to the selected IHC protocol. It cannot be excluded that a markedly less sensitive protocol would have distinguished a subgroup of tumors with a particularly high MRE11 expression among our 3+ positive tumors and that these tumors could be characterized by aggressive behavior. The results of several functional studies have indeed indicated associations between aggressive cancer cell properties and high MRE11 expression. In breast cancer cells, MRE11 overexpression resulted in increased cell proliferation through STAT3 activation, and enhanced tumor cell invasion and migration through activation of MMP-2 and MMP-9 secretion [11]. In oral squamous cancer cells, high MRE11 expression promoted high cell proliferation, migration and invasion via a nuclease-independent pathway and conferred radiotherapy resistance through a nuclease-dependent mechanism while its knockdown increased sensitivity to radiotherapy [41].

The main limitation of our study is the still low number of cases. By using the TMA approach, it would be possible to analyze three to four times more cases without substantial increase in the manpower and reagents needed for tumor analysis. Higher number of cases would enable us to obtain markedly stronger statistical data which is also desirable in the context of the high number of statistical analyses in the present study. The absence of treatment information did not allow us to estimate the impact of treatment strategies on MRE11 expression. Future studies should clarify this point.

## 5. Conclusions

Our data show that MRE11 expression is abundant in most cancer cells, that a reduced expression is associated with an aggressive phenotype in several cancer types and that a complete MRE11 expression loss (MRE11 deficiency) occurs in a very small but distinct proportion of cancers. The potential to exploit MRE11 deficiency as a target for synthetic lethality needs to be further explored.

## Figures and Tables

**Table 1:** MRE11 immunostaining in human tumors.

			MRE11 Immunostaining				
	Tumor Entity	on TMA (n)	Analyzable (n)	Negative (%)	Weak (%)	Moderate (%)	Strong (%)
**Tumors of the skin**	Basal cell carcinoma of the skin	41	33	0.0	0.0	0.0	100.0
	Squamous cell carcinoma of the skin	95	92	0.0	0.0	5.4	94.6
	Malignant melanoma	19	17	0.0	0.0	0.0	100.0
	Malignant melanoma lymph node metastasis	86	73	0.0	1.4	9.6	89.0
	Merkel cell carcinoma	2	1	0.0	0.0	0.0	100.0
**Tumors of the head and neck**	Squamous cell carcinoma of the larynx	109	83	0.0	0.0	1.2	98.8
	Squamous cell carcinoma of the pharynx	60	56	0.0	0.0	5.4	94.6
	Oral squamous cell carcinoma (floor of the mouth)	130	115	0.0	0.9	0.0	99.1
	Pleomorphic adenoma of the parotid gland	50	45	0.0	0.0	0.0	100.0
	Warthin tumor of the parotid gland	49	46	0.0	0.0	2.2	97.8
	Basal cell adenoma of the salivary gland	15	15	0.0	0.0	0.0	100.0
**Tumors of the lung, pleura and thymus**	Adenocarcinoma of the lung	196	165	1.2	0.6	11.5	86.7
	Squamous cell carcinoma of the lung	80	70	0.0	0.0	8.6	91.4
	Mesothelioma, epithelioid	40	26	0.0	0.0	7.7	92.3
	Mesothelioma, biphasic	29	20	0.0	0.0	0.0	100.0
	Thymoma	29	26	0.0	0.0	0.0	100.0
	Lung, neuroendocrine tumor (NET)	29	26	0.0	0.0	3.8	96.2
**Tumors of the female genital tract**	Squamous cell carcinoma of the vagina	30	29	0.0	0.0	10.3	89.7
	Squamous cell carcinoma of the vulva	107	101	0.0	0.0	4.0	96.0
	Squamous cell carcinoma of the cervix	88	88	0.0	1.1	13.6	85.2
	Adenocarcinoma of the cervix	23	23	0.0	0.0	0.0	100.0
	Endometrioid endometrial carcinoma	288	268	1.9	1.1	6.0	91.0
	Endometrial serous carcinoma	36	31	0.0	0.0	0.0	100.0
	Carcinosarcoma of the uterus	57	55	0.0	1.8	21.8	76.4
	Endometrial carcinoma, high grade, G3	13	13	7.7	0.0	0.0	92.3
	Endometrial clear cell carcinoma	9	8	0.0	0.0	25.0	75.0
	Endometrioid carcinoma of the ovary	93	79	0.0	0.0	6.3	93.7
	Serous carcinoma of the ovary	530	446	0.2	0.0	4.0	95.7
	Mucinous carcinoma of the ovary	75	57	0.0	0.0	7.0	93.0
	Clear cell carcinoma of the ovary	51	44	0.0	2.3	18.2	79.5
	Carcinosarcoma of the ovary	47	43	2.3	2.3	7.0	88.4
	Granulosa cell tumor of the ovary	44	38	0.0	0.0	0.0	100.0
	Leydig cell tumor of the ovary	4	4	0.0	0.0	0.0	100.0
	Sertoli cell tumor of the ovary	1	1	0.0	0.0	0.0	100.0
	Sertoli Leydig cell tumor of the ovary	3	3	0.0	0.0	0.0	100.0
	Steroid cell tumor of the ovary	3	3	0.0	0.0	0.0	100.0
	Brenner tumor	32	26	0.0	0.0	3.8	96.2
**Tumors of the breast**	Invasive breast carcinoma of no special type	499	473	0.0	0.0	6.8	93.2
	Lobular carcinoma of the breast	150	135	0.0	0.7	5.2	94.1
	Medullary carcinoma of the breast	8	8	0.0	0.0	25.0	75.0
	Tubular carcinoma of the breast	2	2	0.0	0.0	0.0	100.0
	Mucinous carcinoma of the breast	7	6	0.0	0.0	16.7	83.3
**Tumors of the digestive system**	Adenomatous polyp, low-grade dysplasia	50	34	0.0	0.0	0.0	100.0
	Adenomatous polyp, high-grade dysplasia	50	41	0.0	0.0	0.0	100.0
	Adenocarcinoma of the colon	2483	2183	0.7	0.5	3.5	95.3
	Gastric adenocarcinoma, diffuse type	215	203	0.0	0.0	3.0	97.0
	Gastric adenocarcinoma, intestinal type	215	208	1.9	0.5	5.8	91.8
	Gastric adenocarcinoma, mixed type	62	58	0.0	1.7	3.4	94.8
	Adenocarcinoma of the esophagus	83	77	0.0	0.0	1.3	98.7
	Squamous cell carcinoma of the esophagus	76	69	0.0	0.0	1.4	98.6
	Squamous cell carcinoma of the anal canal	91	68	0.0	0.0	7.4	92.6
	Cholangiocarcinoma	168	148	0.7	0.0	6.8	92.6
	Gallbladder adenocarcinoma	51	47	0.0	0.0	6.4	93.6
	Gallbladder Klatskin tumor	42	32	0.0	0.0	6.3	93.8
	Hepatocellular carcinoma	202	193	4.7	5.2	30.6	59.6
	Ductal adenocarcinoma of the pancreas	659	589	0.2	0.3	6.6	92.9
	Pancreatic/Ampullary adenocarcinoma	98	91	1.1	0.0	9.9	89.0
	Acinar cell carcinoma of the pancreas	18	17	0.0	0.0	17.6	82.4
	Gastrointestinal stromal tumor (GIST)	62	59	0.0	0.0	0.0	100.0
	Appendix, neuroendocrine tumor (NET)	25	16	0.0	0.0	6.3	93.8
	Colorectal, neuroendocrine tumor (NET)	12	8	0.0	0.0	25.0	75.0
	Ileum, neuroendocrine tumor (NET)	53	49	0.0	0.0	20.4	79.6
	Pancreas, neuroendocrine tumor (NET)	101	89	0.0	0.0	6.7	93.3
	Colorectal, neuroendocrine carcinoma (NEC)	14	12	0.0	0.0	0.0	100.0
	Ileum, neuroendocrine carcinoma (NEC)	8	7	0.0	0.0	0.0	100.0
	Gallbladder, neuroendocrine carcinoma (NEC)	4	4	0.0	0.0	0.0	100.0
	Pancreas, neuroendocrine carcinoma (NEC)	14	14	0.0	0.0	7.1	92.9
**Tumors of the urinary system**	Non-invasive papillary urothelial carcinoma, pTa G2 low grade	87	77	0.0	0.0	2.6	97.4
	Non-invasive papillary urothelial carcinoma, pTa G2 high grade	80	67	1.5	0.0	1.5	97.0
	Non-invasive papillary urothelial carcinoma, pTa G3	126	100	0.0	0.0	0.0	100.0
	Urothelial carcinoma, pT2-4 G3	735	565	0.2	0.2	3.0	96.6
	Squamous cell carcinoma of the bladder	22	19	0.0	0.0	0.0	100.0
	Small cell neuroendocrine carcinoma of the bladder	5	5	0.0	0.0	0.0	100.0
	Sarcomatoid urothelial carcinoma	25	19	0.0	0.0	0.0	100.0
	Urothelial carcinoma of the kidney pelvis	62	55	0.0	0.0	7.3	92.7
	Clear cell renal cell carcinoma	1287	1011	0.7	2.4	25.0	71.9
	Papillary renal cell carcinoma	368	289	0.0	2.1	9.0	88.9
	Clear cell (tubulo) papillary renal cell carcinoma	26	16	0.0	0.0	18.8	81.3
	Chromophobe renal cell carcinoma	170	133	0.0	1.5	14.3	84.2
	Oncocytoma of the kidney	257	199	0.0	0.0	2.5	97.5
**Tumors of the male genital organs**	Adenocarcinoma of the prostate, Gleason 3 + 3	83	72	0.0	0.0	13.9	86.1
	Adenocarcinoma of the prostate, Gleason 4 + 4	80	72	0.0	0.0	1.4	98.6
	Adenocarcinoma of the prostate, Gleason 5 + 5	85	82	0.0	1.2	1.2	97.6
	Adenocarcinoma of the prostate (recurrence)	258	222	0.0	0.0	0.5	99.5
	Small cell neuroendocrine carcinoma of the prostate	2	1	0.0	0.0	0.0	100.0
	Seminoma	682	597	0.0	0.8	20.6	78.6
	Embryonal carcinoma of the testis	54	31	0.0	0.0	32.3	67.7
	Leydig cell tumor of the testis	31	29	0.0	0.0	0.0	100.0
	Sertoli cell tumor of the testis	2	2	0.0	0.0	0.0	100.0
	Sex cord stromal tumor of the testis	1	1	0.0	0.0	0.0	100.0
	Spermatocytic tumor of the testis	1	1	0.0	0.0	0.0	100.0
	Yolk sac tumor	53	37	0.0	5.4	21.6	73.0
	Teratoma	53	29	0.0	0.0	3.4	96.6
	Squamous cell carcinoma of the penis	92	89	0.0	0.0	4.5	95.5
**Tumors of endocrine organs**	Adenoma of the thyroid gland	63	62	0.0	0.0	0.0	100.0
	Papillary thyroid carcinoma	341	326	0.0	0.0	3.7	96.3
	Follicular thyroid carcinoma	109	103	0.0	0.0	3.9	96.1
	Medullary thyroid carcinoma	57	54	0.0	0.0	1.9	98.1
	Parathyroid gland adenoma	43	37	0.0	0.0	0.0	100.0
	Anaplastic thyroid carcinoma	19	19	5.3	0.0	10.5	84.2
	Adrenal cortical adenoma	48	39	0.0	2.6	28.2	69.2
	Adrenal cortical carcinoma	27	25	0.0	0.0	12.0	88.0
	Pheochromocytoma	51	45	2.2	4.4	15.6	77.8
**Tumors of hematopoetic and lymphoid tissues**	Hodgkin’s lymphoma	103	88	0.0	0.0	1.1	98.9
	Small lymphocytic lymphoma, B-cell type (B-SLL/B-CLL)	50	48	0.0	0.0	6.3	93.8
	Diffuse large B cell lymphoma (DLBCL)	113	112	0.0	0.9	8.0	91.1
	Follicular lymphoma	88	87	1.1	0.0	6.9	92.0
	T-cell non-Hodgkin’s lymphoma	25	25	0.0	4.0	0.0	96.0
	Mantle cell lymphoma	18	18	0.0	5.6	0.0	94.4
	Marginal zone lymphoma	16	15	0.0	0.0	6.7	93.3
	Diffuse large B-cell lymphoma (DLBCL) in the testis	16	16	0.0	0.0	0.0	100.0
	Burkitt lymphoma	5	3	0.0	0.0	33.3	66.7
**Tumors of soft tissue and bone**	Granular cell tumor	23	14	0.0	0.0	7.1	92.9
	Leiomyoma	50	45	0.0	0.0	11.1	88.9
	Leiomyosarcoma	94	84	0.0	2.4	19.0	78.6
	Liposarcoma	96	87	0.0	1.1	4.6	94.3
	Malignant peripheral nerve sheath tumor (MPNST)	15	15	0.0	0.0	13.3	86.7
	Myofibrosarcoma	26	26	0.0	0.0	0.0	100.0
	Angiosarcoma	42	31	0.0	0.0	0.0	100.0
	Angiomyolipoma	91	72	0.0	1.4	11.1	87.5
	Dermatofibrosarcoma protuberans	21	15	0.0	0.0	0.0	100.0
	Ganglioneuroma	14	13	0.0	0.0	0.0	100.0
	Kaposi sarcoma	8	4	0.0	0.0	0.0	100.0
	Neurofibroma	117	105	0.0	0.0	1.0	99.0
	Sarcoma, not otherwise specified (NOS)	74	66	0.0	0.0	7.6	92.4
	Paraganglioma	41	39	2.6	0.0	2.6	94.9
	Ewing sarcoma	23	12	0.0	0.0	0.0	100.0
	Rhabdomyosarcoma	7	6	0.0	0.0	0.0	100.0
	Schwannoma	122	117	0.0	0.0	0.0	100.0
	Synovial sarcoma	12	10	0.0	0.0	0.0	100.0
	Osteosarcoma	19	10	0.0	0.0	10.0	90.0
	Chondrosarcoma	15	12	0.0	0.0	8.3	91.7
	Rhabdoid tumor	5	5	0.0	0.0	0.0	100.0
	Solitary fibrous tumor	17	17	0.0	0.0	0.0	100.0

**Table 2:** MRE11 immunostaining and tumor phenotype.

		n	Low to Intermediate Positive (%)	Strongly Positive (%)	*p*
Invasive breast carcinoma of no special type	All tumors	400	6.5	93.5	
	pT1	166	4.8	95.2	0.3995
	pT2	193	8.3	91.7	
	pT3-4	36	5.6	94.4	
	G1	14	0.0	100.0	0.2886
	G2	225	7.6	92.4	
	G3	160	5.6	94.4	
	pN0	198	6.6	93.4	0.6803
	pN+	163	5.5	94.5	
Serous carcinoma of the ovary	pT1	30	0.0	100.0	0.0396
	pT2	41	0.0	100.0	
	pT3	236	5.1	94.9	
	pN0	80	2.5	97.5	0.3925
	pN+	148	4.7	95.3	
Clear cell renal cell carcinoma	All tumors	975	27.9	72.1	
	ISUP 1	227	23.8	76.2	0.0249
	ISUP 2	331	26.6	73.4	
	ISUP 3	227	36.1	63.9	
	ISUP 4	68	27.9	72.1	
	Fuhrman 1	53	11.3	88.7	0.0015
	Fuhrman 2	573	26.7	73.3	
	Fuhrman 3	253	35.2	64.8	
	Fuhrman 4	80	27.5	72.5	
	Thoenes 1	305	23.0	77.0	0.0028
	Thoenes 2	408	33.3	66.7	
	Thoenes 3	86	37.2	62.8	
	UICC 1	295	23.7	76.3	0.0014
	UICC 2	31	41.9	58.1	
	UICC 3	83	43.4	56.6	
	UICC 4	60	36.7	63.3	
	pT1	583	20.1	79.9	<0.0001
	pT2	113	44.2	55.8	
	pT3-4	266	38.7	61.3	
	pN0	153	35.3	64.7	0.8510
	pN+	24	33.3	66.7	
	M0	97	33.0	67.0	0.8907
	M+	75	32.0	68.0	
Papillary renal cell carcinoma	All tumors	258	10.9	89.1	
	ISUP 1	38	7.9	92.1	0.6038
	ISUP 2	116	12.1	87.9	
	ISUP 3	72	12.5	87.5	
	ISUP 4	5	0.0	100.0	
	Fuhrman 1	3	0.0	100.0	0.3867
	Fuhrman 2	164	11.0	89.0	
	Fuhrman 3	70	12.9	87.1	
	Fuhrman 4	9	0.0	100.0	
	Thoenes 1	53	11.3	88.7	0.9777
	Thoenes 2	143	11.9	88.1	
	Thoenes 3	15	13.3	86.7	
	UICC 1	92	12.0	88.0	0.1880
	UICC 2	17	0.0	100.0	
	UICC 3	4	0.0	100.0	
	UICC 4	10	10.0	90.0	
	pT1	181	8.8	91.2	0.1836
	pT2	44	15.9	84.1	
	pT3-4	26	19.2	80.8	
	pN0	21	14.3	85.7	0.6053
	pN+	12	8.3	91.7	
Hepatocellular carcinoma	All tumors	231	26.4	73.6	
	pT1	72	30.6	69.4	0.7150
	pT2	81	24.7	75.3	
	pT3-4	60	26.7	73.3	
	pN0	71	26.8	73.2	0.0248
	pN+	41	9.8	90.2	
	G 1	34	38.2	61.8	0.2975
	G 2	126	24.6	75.4	
	G 3	50	26.0	74.0	
Ductal adenocarcinoma of the pancreas	All tumors	477	7.3	92.7	
	pT1	10	10.0	90.0	0.7027
	pT2	65	4.6	95.4	
	pT3	373	7.5	92.5	
	pT4	27	11.1	88.9	
	G1	14	0.0	100.0	0.2435
	G2	337	7.4	92.6	
	G3	103	9.7	90.3	
	pN0	102	9.8	90.2	0.3068
	pN+	372	6.7	93.3	
	R0	238	7.1	92.9	0.6294
	R1	203	8.4	91.6	
	All tumors	386	6.2	93.8	
Adenocarcinoma of the stomach	pT1-2	58	3.4	96.6	0.4505
	pT3	124	6.5	93.5	
	pT4	122	8.2	91.8	
	pN0	82	9.8	90.2	0.1941
	pN+	221	5.4	94.6	
	MMR proficient	256	3.1	96.9	<0.0001
	MMR deficient	41	22.0	78.0	
Papillary thyroid carcinoma	pT1	149	2.0	98.0	0.1218
	pT2	78	7.7	92.3	
	pT3-4	97	3.1	96.9	
	pN0	90	0.0	100.0	0.0332
	pN+	120	3.3	96.7	
Endometrioid endometrial carcinoma	All tumors	166	6.6	93.4	
	pT1	103	3.9	96.1	0.1086
	pT2	24	16.7	83.3	
	pT3-4	36	8.3	91.7	
	pN0	50	6.0	94.0	0.5170
	pN+	30	10.0	90.0	
Seminoma of the testis	All tumors	525	21.5	78.5	
	pT1	337	16.9	83.1	0.0003
	pT2	130	34.6	65.4	
	pT3	51	19.6	80.4	
	Haemangio invasion				
	negative	427	21.3	78.7	0.5967
	positive	53	24.5	75.5	
	Lymphangio invasion				
	negative	375	18.4	81.6	0.0019
	positive	110	32.7	67.3	
	Infiltration of the spermatic cord negative	407	21.9	78.1	0.8206
	positive	56	23.2	76.8	
	Infiltration Rete Testis				
	negative	227	18.9	81.1	0.1060
	positive	264	25.0	75.0	
Colorectal adenocarcinoma	All tumors	2109	4.7	95.3	
	pT1	81	1.2	98.8	0.0555
	pT2	406	3.4	96.6	
	pT3	1173	4.9	95.1	
	pT4	410	6.6	93.4	
	pN0	1071	5.2	94.8	0.4070
	pN+	990	4.4	95.6	
	V0	1490	5.3	94.7	0.1351
	V1	537	3.7	96.3	
	L0	672	5.1	94.9	0.8041
	L1	1373	4.8	95.2	
	right side	423	9.2	90.8	<0.0001
	left side	1123	2.6	97.4	
	MMR proficient	1082	2.3	97.7	<0.0001
	MMR deficient	78	25.6	74.4	

Abbreviations: pT: pathological tumor stage, G: grade, pN: pathological lymph node status, M: status of distant metastasis, V: venous invasion, L: lymphatic invasion, R: resection status, MMR: mismatch repair, ISUP: International Society of Urological Pathology, UICC: Union for International Cancer Control.

## Data Availability

The original contributions presented in this study are included in the article/Supplementary Material. Further inquiries can be directed to the corresponding author.

## Supplementary Materials

The following supporting information can be downloaded at https://www.mdpi.com/article/10.3390/diagnostics16131965/s1, Supplementary Figure S1: Antibody validation by comparison of antibodies. Supplementary Figure S2: Comparison of study data with previous reports on MRE11 IHC.
