# Supplementary figures and images for: MRE11 Deficiency Occurs in a Small Group of Cancers from Various Different Tumor Entities
Source: Diagnostics (Basel). 2026 Jun 24;16(13):1965. doi: 10.3390/diagnostics16131965 (PMC13360613; doi:10.3390/diagnostics16131965)

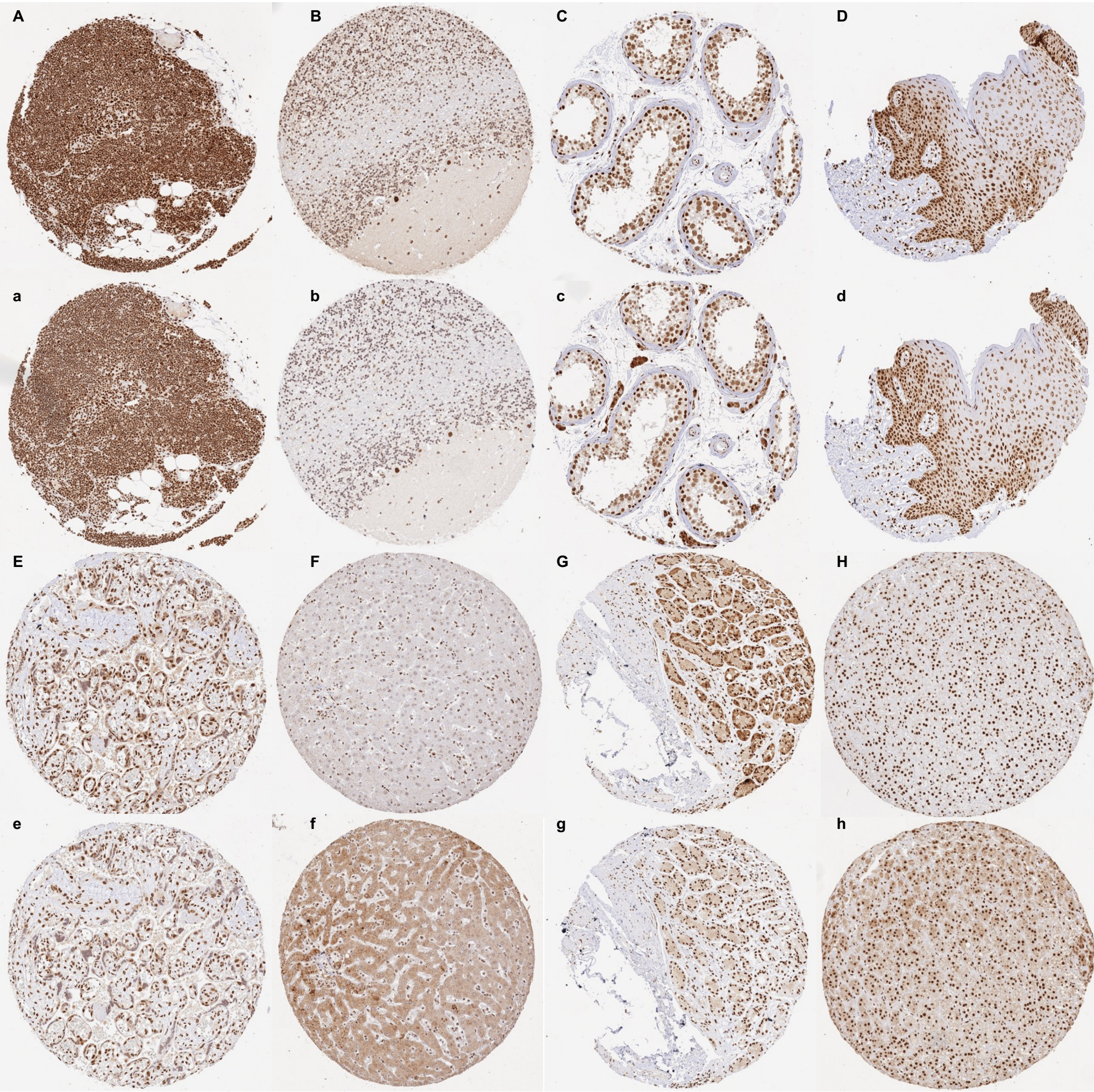

Supplement: Supplementary file 1 [file diagnostics-16-01965-s001.zip › Suppl Figure 1 MRE11 MTA.pdf]

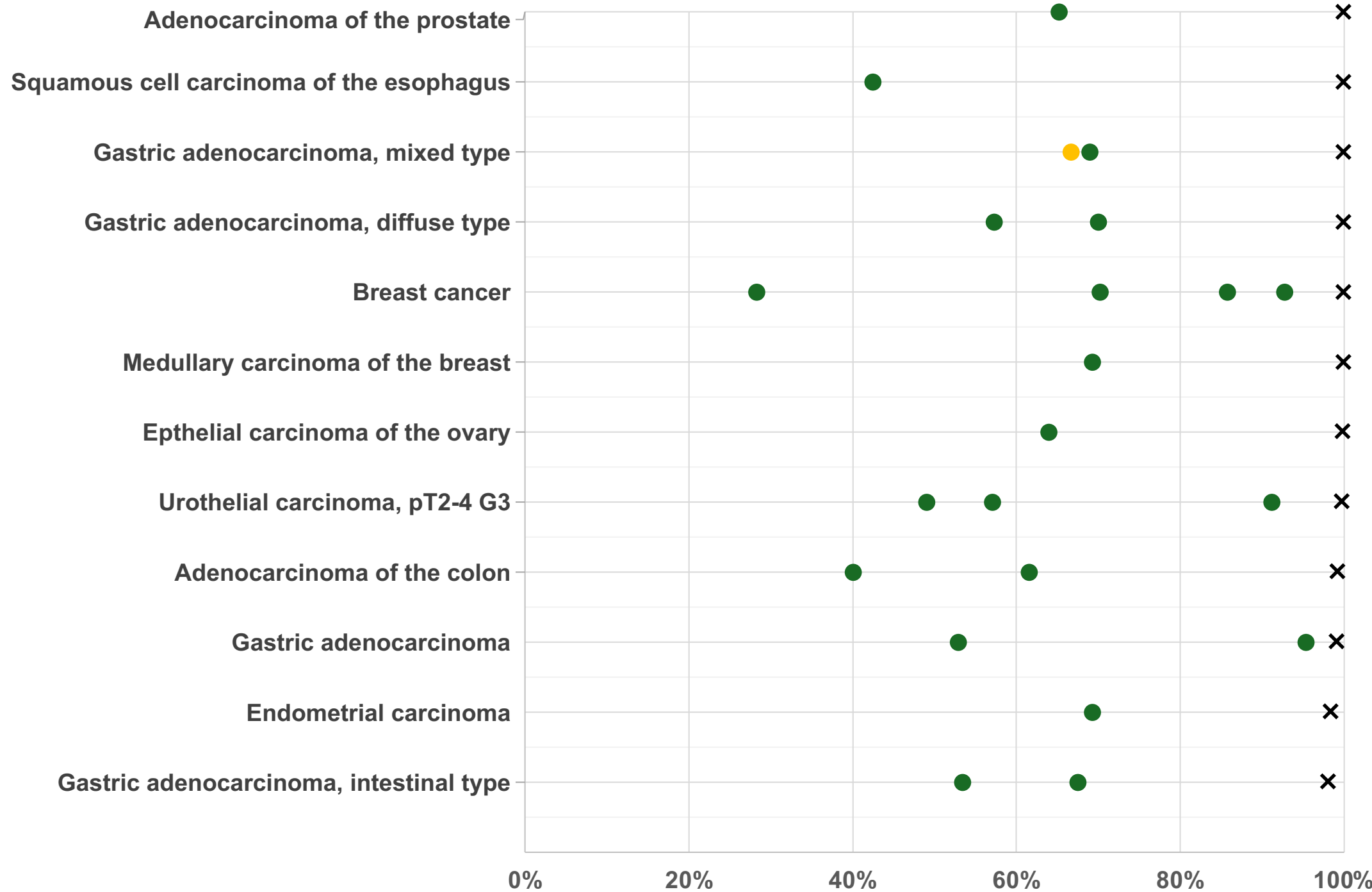

Supplement: Supplementary file 1 [file diagnostics-16-01965-s001.zip › Suppl Figure 2 MRE11 MTA.pdf]
